# An Aminoglycoside-Sparing Regimen with Double Beta-Lactam to Successfully Treat *Granulicatella adiacens* Prosthetic Aortic Valve Endocarditis—Time to Change Paradigm?

**DOI:** 10.3390/idr16020020

**Published:** 2024-03-14

**Authors:** Alberto Pagotto, Floriana Campanile, Paola Conti, Francesca Prataviera, Paola Della Siega, Sarah Flammini, Simone Giuliano, Luca Martini, Davide Pecori, Assunta Sartor, Maria Screm, Tosca Semenzin, Carlo Tascini

**Affiliations:** 1 Infectious Diseases Division, Department of Medicine (DAME), Azienda Sanitaria Universitaria Friuli Centrale (ASUFC), 33100 Udine, Italypaola.dellasiega@asufc.sanita.fvg.it (P.D.S.); sarah.flammini@asufc.sanita.fvg.it (S.F.); simone.giuliano@asufc.sanita.fvg.it (S.G.); lucamartini9@gmail.com (L.M.); davide.pecori@asufc.sanita.fvg.it (D.P.); tosca.semenzin@gmail.com (T.S.); carlo.tascini@asufc.sanita.fvg.it (C.T.); 2 Department of Biomedical and Biotechnological Sciences, Section of Microbiology, University of Catania, 95123 Catania, Italy; f.campanile@unict.it (F.C.); paolacontias@hotmail.com (P.C.); 3 Microbiology Unit, Azienda Sanitaria Universitaria Friuli Centrale (ASUFC), 33100 Udine, Italy; assunta.sartor@asufc.sanita.fvg.it (A.S.); maria.screm@asufc.sanita.fvg.it (M.S.)

**Keywords:** nutritionally variant streptococci, *Granulicatella* spp., infective endocarditis, penicillin-binding proteins, antibiotics, betalactams

## Abstract

(1) Background: *Granulicatella adiacens* is a former nutritionally variant streptococci (NVS). NVS infective endocarditis (IE) is generally characterized by a higher rate of morbidity and mortality, partially due to difficulties in choosing the most adequate microbiological culture method and the most effective treatment strategy, and partially due to higher rates of complications, such as heart failure, peripheral septic embolism, and peri-valvular abscess, as well as a higher rate of valve replacement. Depending on the affected valve (native valve endocarditisNVE, or prosthetic valve endocarditisPVE), the American Heart Association (AHA) 2015 treatment guidelines (GLs) suggest penicillin G, ampicillin, or ceftriaxone *plus* gentamicin (2 weeks for NVE and up to 6 weeks for PVE), while vancomycin alone may be a reasonable alternative in patients who are intolerant of β-lactam therapy. The European Society of Cardiology (ESC) 2023 GLs recommend treating NVE with penicillin G, ceftriaxone, or vancomycin for 6 weeks, suggesting combined with an aminoglycoside (AG) for at least the first 2 weeks only for PVE; likewise, the same recommendations for IE due to Enterococcus faecalis. (2) Methods: Starting from the case of a 51-year-old man with *G. adiacens* aortic bio-prosthesis IE who was successfully treated with aortic valve replacement combined with double beta-lactams, an AG-sparing regimen, we performed microbiology tests in order to validate this potential treatment change. (3) Results: As for *E. faecalis* IE, we found that the combination of ampicillin *plus* cephalosporines (like ceftriaxone or ceftobiprole) showed a synergistic effect in vitro, probably due to wider binding to penicillin-binding proteins (PBPs), thus contributing to enhanced bacterial killing and good clinical outcome, as well as avoiding the risk of nephrotoxicity due to AG association therapy. (4) Conclusions: Further studies are required to confirm this hypothesis, but double beta-lactams and an adequate sourcecontrol could be a choice in treating *G. adiacens* IE.

## 1. Introduction

Infective endocarditis (IE) remains a severe disease that is associated with high morbidity and mortality, despite many efforts aimed at improving diagnostic and therapeutic strategies. As reported in the European Infective Endocarditis Registry, the most frequent microorganisms identified remain staphylococci in 44.1%, enterococci in 15.8%, oral streptococci in 12.4%, and *Streptococcus gallolyticus* in 6.6% [1]. *Granulicatella* spp., previously known as NVS, are considered (together with *Abiotrophia* spp.) to be responsible for 5% of all streptococcal endocarditis. This fastidious bacterium has been reported to be a common cause of culture-negative bacterial endocarditis and its role in this scenario could be underestimated [2]. *Granulicatella* is catalase-negative, oxidase-negative, facultatively anaerobic, Gram-positive coccus, generally found as part of the normal oral, genitourinary, and intestinal tracts’ flora, with the oral cavity being the most probable bacterial entry point for most cases of IE [3,4]. Although its role as a causative agent of IE is known, diagnostic and clinical management are not completely defined. Depending on the affected valve (native or prosthetic), international GLs suggest treating *G. adiacens* IE with a combination therapy of beta-lactam *plus* gentamicin for 2 up to 6 weeks (with different classes and/or levels of recommendation), as in enterococcal endocarditis [5,6]. As reported in other experience [7], we tried to confirm that intravenous double beta-lactam combination therapy could result in good outcomes also for IE due to *G. adiacens*.

## 2. Case Presentation

A 51-year-old man with a dual-chamber pacemaker (PM Ensura DDDR) implanted in March 2017 due to a Bruce treadmill test revealing 1° atrio-ventricular block and iso-rhythmic atrio-ventricular dissociation, an aortic valve bio-prosthesis (Carpentier-Edwards Perimount Magna EASE 25 mm), *plus* an ascending aorta vascular prosthesis (Vascutek Gelweave 28 mm), both implanted in September 2017 due to severe aortic valve stenosis in the bicuspid valve and ascending aorta ectasia, was admitted in August 2022 to the emergency department (ED) of Udine’s Hospital (Friuli Venezia-Giulia Region, Italy), with fever (up to 38 °C) for about 2 weeks and dry cough (lasting for 2 months after COVID-19 infection occurred in June 2022). He also complained of lumbar pain and a history of lumbar disc herniation, with no relief after taking anti-inflammatory and muscle relaxant drugs for at least 2 weeks. He denied any dental procedures and/or urinary tract or abdominal problems in the period before hospital admission. Physical examination showed oxygen blood saturation of 97%, bilateral norm-transmitted vesicular murmur with no other respiratory findings (no rales, no whistles, no crackles), preserved and rhythmic cardiac activity with controlled heart rate, a low-moderate systolic murmur (2/6) at the aortic focus, irradiated to the neck, with good hemodynamic stability (no peripheral edema), nor abdominal, neurological, or cutaneous pathological findings.

Initial laboratory studies revealed a C-reactive protein (CRP) value of 51.12 mg/L (cut-off 0–5 mg/L) and procalcitonin value of 0.12 ng/mL (cut-off < 0.10 ng/mL). The transthoracic echocardiography (TTE) performed in the emergency room revealed a vegetation with a thickening of the aortic bio-prosthesis (not seen in a previous recent control). Blood cultures (four bottles) were taken and, after one day, all samples yielded Gram-positive cocci in chains subsequently identified as *Granulicatella adiacens* by Bruker MALDI-TOF at the microbiology department of Udine’s Hospital. Antimicrobial susceptibility testing, conducted on the Evo Freedom Tecan workstation using the commercial MICRONAUT-S panel (MERLIN Diagnostika GmbH, Bornheim-Hersel, Germany) by micro-dilution broth method, showed susceptibility to amoxicillin (≤0.016 mg/L); cefotaxime (0.125 mg/L), ceftriaxone (0.094 mg/L), gentamicin (0.38 mg/L), meropenem (0.125 mg/L), and penicillin G (0.125 mg/L), according to standard guidelines.

A total body computed tomography (CT) scan was performed, which did not reveal any secondary embolism (including no sign of spondyliscitis), nor the dental scan any signs of peri-apical radiolucency. A second echocardiography control performed 1 week after hospital admission (both transthoracic and transesophageal) confirmed a vegetation both on the aortic right coronary cusp (14 mm × 6 mm) and on the non-coronary cusp with minimum regurgitation; no apparent peri-valvular abscess, no vegetation on PM electrodeleads, and no other valve infectious involvement was observed. 

While waiting for blood culture results, an empiric iv therapy with daptomycin (10 mg/kg/day) and ceftaroline (600 mg tid) was started. After *G. adiacens* was identified (4 days after blood samples were taken), antibiotic therapy was switched to 4 g ampicillin intravenously every 6 h in continuous infusion (optimal dosage driven by therapeutic drug monitoring) and 2 g ceftriaxone intravenously every 12 h. This regimen was selected in order to avoid AG nephrotoxicity, although the patient’s serum creatinine levels were normal. 

The case was carefully discussed with the cardiac surgeon team and, considering the fastidious isolated bacteria, the vegetation dimensions (near 15 mm) with high risk of embolism, the presence of prosthetic devices, and supposed low intra-operative and post-operative risks (young patient with no other comorbidity and with long life expectancy), the patient underwent surgery 2 days after starting the new antibiotic regimen. The intra-operative field revealed large masses attached to the previous implanted bio-prosthesis (almost on the ventricular side) but no abscess cavity; furthermore, the wall of the aortic bulb appeared thinned and fragile, while the valvular anulus was really damaged and discontinuous, especially under the coronary *ostia*. Therefore, surgeons decided to perform aortic valve bio-prosthesis and ascending aorta prosthesis replacement with a biological stentless root (Medtronic Freestyle 25 mm), an aortic vascular prosthesis (Vascutek Gelweave 28 mm), *plus* PM and leads removal. Shortly afterward (5 days after major surgery), the patient underwent PM re-implantation (with bi-cameral Medtronic Azure S DR). The standard culture results of the intra-operative samples (vegetation, aortic valve bio-prosthesis, aortic vascular prosthesis) were negative. Unfortunately, PM leads culture and valve histopathology were not performed. 

Intravenous antibiotic therapy was continued for overall 6 weeks after first blood culture negativization (this occurred 10 days after first positivization), then the patient was discharged from the hospital with oral therapy (amoxicillin plus cefditoren pivoxil). Far from any GL recommendations about the correct timing of antibiotic treatment in NVS PVE, we decided to continue home treatment in order to avoid any risk of possible relapse considering that *Granulicatella* spp. are fastidious bacteria that are very hard to eradicate, even in cases where the strain is highly susceptible, the intra-operative field revealed a very complex and fragile situation with the necessity of both aortic valve and ascending aorta replacement, and the patient was fairly young with a history of re-operation. 

Three weeks later, during a follow-up ambulatory visit, there was no clinical nor microbiological evidence of relapse, so antibiotics were stopped. The last TTE control performed 10 months after aortic valve replacement revealed no signs of IE relapse. 

## 3. Laboratory Tests

In order to validate the potential synergism by using double beta-lactams in *Granulicatella* spp. treatment, the bacterial strain was sent to a reference microbiology laboratory at Catania University (Italy). 

**Figure 1:**
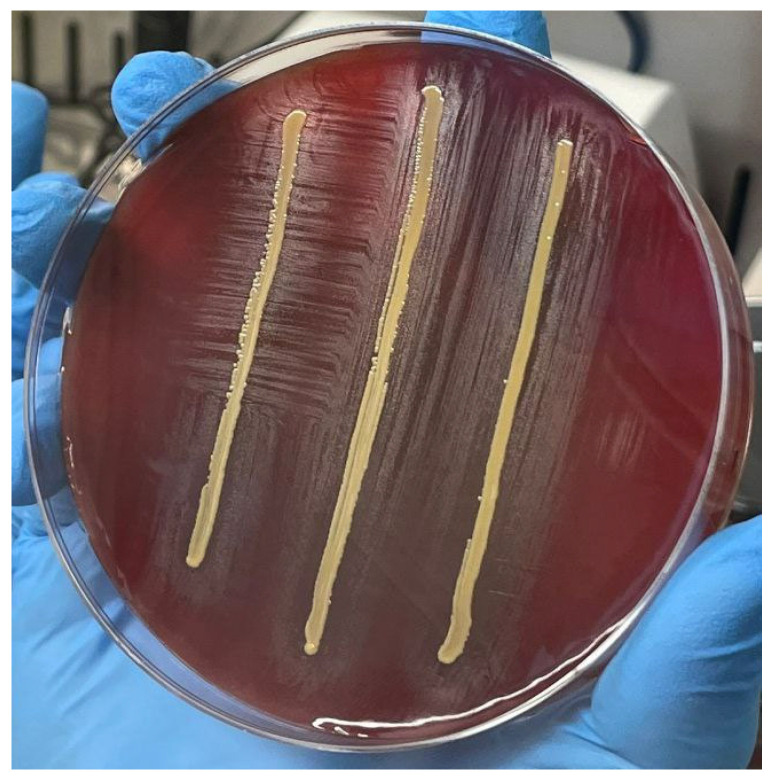
Satellite growth of *Granulicatella adiacens* in proximity to the staphylococcal streak.

The identification was carried out by sequencing an internal fragment of the 16S rRNA gene, as already described [8]. Sequence alignment was performed using BLAST (Basic Local Alignment Search Tool; https://blast.ncbi.nlm.nih.gov/Blast.cgi accessed on 4 September 2023), which identified *G. adiacens* (100% NT identity with *G. adiacens* ATCC 49175 ID: CP102283.1). The organism was co-cultivated on blood agar with a streak of *Staphylococcus aureus* to demonstrate satellitism, which is a distinctive trait of this organism (Figure 1).

Antibiotic susceptibility testing was performed using Minimum Inhibitory Concentration (MIC) Test Strips (Liofilchem ^®^, Roseto degli Abruzzi, Italy) on in-house chocolate agar using Columbia Blood Agar Base (Oxoid, Milan, Italy) supplemented with 5% defibrinated horse blood (Biolife Italiana S.r.l., Milan, Italy) and 0.01% L-cysteine (100 mcg/mL) (Sigma-Aldrich^®^, Louis, MO, USA). Tests were performed in duplicate. MICs were interpreted using the CLSI M45 breakpoints for *Abiotrophia* spp. and *Granulicatella* spp. when available (CLSI 2015. Methods for antimicrobial dilution and disk susceptibility testing of infrequently isolated or fastidious bacteria, 3rd ed, CLSI guideline M45. Clinical and Laboratory Standards Institute, Wayne, PA). For quality control, we used CLSI-recommended *Streptococcus pneumoniae* ATCC 49619. According to CLSI guidelines, the strain showed resistance to ceftriaxone and cefotaxime, non-susceptibility to penicillin and ampicillin, and susceptibility to imipenem and vancomycin (Table 1).

**Figure 2:**
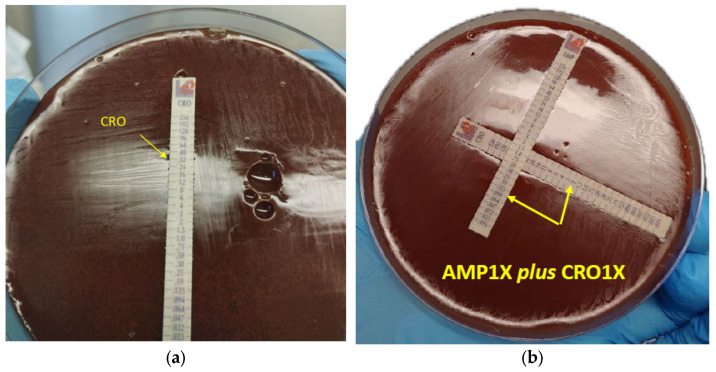
Antimicrobial susceptibility testing and synergistic combination of ceftriaxone (CRO) with ampicillin (AMP) by gradient-cross method. Arrowheads indicate the MIC values of CRO alone (**a**) and in combination (**b**) with AMP (AMP1X plus CRO1X).

Synergy testing was performed in duplicate, using the gradient-cross or 90° angle method, with some modification. Briefly, MIC test strips were placed in a cross formation, with a 90° angle at the intersection between the scales at their respective MICs for the organism, and incubated for 18 h at 35 °C. The following combinations were tested: ampicillin + ceftobiprole (AMP + BPR), ampicillin + ceftriaxone (AMP + CRO), ampicillin + gentamicin (AMP + CN); ceftobiprole + gentamicin (BPR + CN); and ceftriaxone + gentamicin (CRO + CN). In some cases, where the combination with the MICs of respective antibiotics did not exhibit a synergistic effect, a modification was made by testing the cross with the 2 × MIC value (Figure 2). The gradient-cross method was performed in duplicate for all combinations. The fractional inhibitory concentration index (FIC index) was used to interpret the gradient-cross method, as follows: synergy, FIC index ≤ 0.5; additive effect, FIC index 0.5–1; indifference, FIC index 1–4; antagonism, FIC index > 4 [9]. Despite their resistance profiles, double beta-lactam combinations showed better synergistic activity. Synergy was demonstrated at their respective MIC values (1 × MIC) when ampicillin was tested in combination with ceftriaxone (Figure 2b) and ceftobiprole, while beta-lactam combinations with gentamicin showed a synergistic effect only with double MICs of ampicillin, ceftriaxone, and ceftobiprole (2 × MIC), as demonstrated by their FICindexes (Table 2).

## 4. Discussion

NVS are fastidious Gram-positive bacteria, requiring either L-cysteine or pyridoxal-supplemented medium to support growth. Chromosomal DNA-DNA hybridization in 1989, and the more recent 16S rRNA gene sequencing data, led to a reclassification of NVS isolates into two different genera, *Abiotrophia* and *Granulicatella*, which in turn comprise four recognized species: *Abiotrophia defectiva*, *Granulicatella adiacens*, *Granulicatella elegans*, and *Granulicatella balaenopterae* [10]. Penicillin, cephalosporin (ceftriaxone, cefotaxime, cefepime, ceftaroline), carbapenem (meropenem, imipenem), vancomycin, daptomycin, and gentamicin susceptibility consistently varies for *G. adiacens* strains in different studies, probably due to difficult isolation and identification techniques [11,12]. Recent studies have shown that molecular biology techniques, such as 16S rDNA NGS analysis, have higher sensitivity than traditional microbiological methods for detecting IE (either blood and heart valve cultures) caused by fastidious or difficult-to-culture microorganisms like *G. adiacens* [13].

Our blood isolate—which was first tested for antimicrobial susceptibility using the micro-dilution broth method—showed good susceptibility to penicillin, ceftriaxone, and gentamicin. However, due to variability in in vitro penicillin susceptibility and the challenges associated with obtaining dependable antimicrobial susceptibility testing (AST) results for these organisms, mono-therapy with penicillin for *G. adiacens* IE, even at high dose, may result in poor outcomes. As reported by Giuliano et al., such organisms may exhibit a laboratory phenomenon of “penicillin tolerance”: for tolerant strains, it is possible that the minimum bactericidal concentration (MBC) of penicillin exceeds the MIC up to 32-fold [2]. Even rabbit model studies showed the superiority of penicillin plus gentamicin compared to penicillin alone [14].

Another potential explanation of antibiotic treatment failure using penicillin in mono-therapy, thus focusing on the problem of the most proper antimicrobial choice (mono-therapy instead of combination therapy) in treating *Granulicatella* spp. IE (and in general streptoccocal IE) apart from PEN susceptibility, could be related to biofilm formation. As described in recent studies, growing evidence suggests that the ability to produce biofilms may play a major pathogenetic role in supporting microbial adhesion and persistence while protecting bacteria from antimicrobial drugs, and recent results support the knowledge that native valve IE (not only prosthetic valve IE), even due to streptococci (as well as *Staphylococcus aureus* and *E. faecalis*), represents a model of biofilm-related infection which, therefore, could be associated with increased antibiotic tolerance/resistance [15].

Thus, treating susceptible NVS strain (and in general streptococcal strain) IE with beta-lactam mono-therapy solely on the basis of blood culture MIC, without any concern about eventual MBC and/or minimum biofilm inhibitory concentration (MBIC), could lead to microbiological and clinical worsening/failure. Nowadays, there is a lack of data about the diffusion gradient of beta-lactams into the vegetation, and the ratio of antibiotic concentration at steady state between plasma and vegetation cannot be completely determined [16]. Assuming that the ratio of the AUC of antibiotics in tissue to the AUC of the total serum concentration is linked to the ratio of diffusible (supposedly unbound) drug in tissue to the total concentration, most of the currently available studies about antibiotic penetration into heart valves are based upon high performance liquid chromatography (HPLC) methods [17,18]. On the one hand, HPLC has been developed widely in recent years and allows the precise determination of the concentrations of most drugs in various body fluids like blood [19], on the other hand, MALDI MS imaging (MSI) and micro dialysis (MD) techniques could represent better methods for visualizing and quantifying antibiotic penetration into the vegetation [20]. But these tools are still waiting for validation. 

Surely, when surgical intervention is considered a feasible and safe option considering the patient’s underlying conditions, surgical disruption and removal of microbial vegetation can largely remove the biofilm structure, thus improving the patient’s healing, as we decided to perform in our case. But surgery cannot lead to complete eradication of infection in peri-valvular cardiac tissues, unless being extremely demolitive, and that is why it would be necessary to continue highperformance antibiotic treatment, depending on the vegetation/valvular culture sample results.

Specifically, our patient underwent a redo operation, as described in a retrospective German study, performed to evaluate the impact of NVE versus PVE on post-operative outcomes and long-term survival and to identify pre-operative risk factors in a large cohort of 4300 patients with IE, in which the evidence suggested that PVE alone should not be a contraindication for redo operations [21].

The 2012 British Society of Antimicrobial Chemotherapy guidelines recommend 4–6 weeks with benzylpenicillin and gentamicin for the treatment of *Granulicatella* or *Abiotrophia* endocarditis [22].

The 2015 GLs from the AHA recommend therapy with penicillin G, AMP, or CRO plus gentamicin (2 weeks for NVE and up to 6 weeks for PVE), while vancomycin alone may be a reasonable alternative in patients who are intolerant of β-lactam therapy [5]. The ESC 2023 GLs recommend treating NVE with penicillin G, CRO, or vancomycin for 6 weeks, suggesting combined with an AG for at least the first 2 weeks only in the case of PVE [6].

However, even with these recommended treatment regimens, the rates of surgical therapy (27%) and relapse and death (17–27%) remain high. Additionally, relapses may occur frequently, even in cases where the strain is highly susceptible [23,24].

This mirrors the outcomes and recommendations for treating *E. faecalis* IE (EFIE). Experimental studies have been successfully carried out as a way to evaluate the synergism of beta-lactam combination against clinical strains of *E. faecalis*, regardless of their susceptibility to aminoglycosides. Specifically, the mainstay for the synergistic activity of beta-lactam combination could be based on to the differential and complementary saturation of *E. faecalis* PBPs, thus generating the necessary bactericidal effect [25,26,27].

In 1995, for the first time ever, Mainardi and co-workers yielded a demonstration of the synergism between amoxicillin and cefotaxime, through partial saturation of essential PBPs 4 and 5 (primary role in cell growth of *E. faecalis*) by amoxicillin (AML), and also total saturation of non-essential PBPs 2 and 3 (probably engaged in cell wall assembly) by cefotaxime [25]. Thereafter, these first pilot test results represented the backbone for clinical studies focused on establishing the true efficacy of this therapeutic approach in humans with *E. faecalis* IE. In a recent review about the efficacy of AMP plus CRO regimen against EFIE, Marino and colleagues concluded that the epidemiological changes of *E. faecalis* IE, that is the fact that the population is aging and consequently becoming more fragile, and a possible underestimation of treatment sideeffects, above all the higher risk of nephrotoxicity, should compel an ideal shift in antibiotic choice and that the growing body of literature with the combination AMP and CRO appears promising. But in this context, large and high-quality non-inferiority clinical studies are needed to clearly assess the efficacy and safety of double beta-lactam regimens against *E. faecalis* IE instead of AG regimens [26].

In a recent retrospective case series by Giuliano at al., 21 patients with a diagnosis of *E. faecalis* IE or primary or non-primary complicated or uncomplicated bacteremia were treated with AMP plus BPR. The results showed a high clinical success rate of 81% and microbiological cure in 86% of patients. One of the reasons for this treatment strategy was that, unlike CRO, BPR can inhibit non-essential high-molecular-weight enterococcal PBPs and has a higher affinity for the essential PBP4, which is a critical lethal target and the main determinant of beta-lactam susceptibility in *E. faecalis* [27].

Regarding IE caused by streptococci with a PEN MIC between 0.25 and 2 mg/L, no prospective comparative study has ever assessed the advantages of adding AG in this setting. This topic was recently confirmed in a retrospective French study (414 patients with streptococcal IE, mostly viridans group streptoccoci), in which the authors observed that streptococcal IE with AML MIC between 0.25 and 2 mg/L had a higher mortality rate than that with lower MIC, but combination with AG was not associated with better outcome [28]. From the microbiological point of view, even if a PEN MIC of 0.25 to 2 mg/L is similar to the PEN MIC for *E. faecalis*, bacterial tolerance to PEN is uncommon in streptococci (10–20%) as compared to enterococci (75%) [29]. Therefore, in a recent review by D. Lebeaux et al., the authors suggested that AG may not be necessary for streptococcal IE with AML MIC between 0.25 and 0.5 mg/L, and their use should be restricted to streptococcal IE with AML MIC > 0.5 mg/L [30].

As recently described by Khan et al., it is possible that using AMP plus CRO therapy for *G. adiacens* IE, similarly to *E. faecalis*, may result in synergy, increased bacterial killing, and better clinical outcome, without the risk of nephrotoxicity due to AG use. In their study, the authors performed susceptibility testing using E-tests for AMP and CRO alone and in combination at standard concentrations, which demonstrated synergy (based on their FICindex) [7]. 

In order to achieve a better understanding of the potential synergism of using double beta-lactam treatment for *Granulicatella* spp. infections, we sent our bacterial strain to a reference microbiology laboratory at Catania University. After identifying the strain as *G. adiacens* through 16S rRNA gene sequencing, it was difficult to perform antimicrobial susceptibility by the recommended BMD method due to growth issues [31]. As there are no commercially available methods with FDA clearance for AST of these fastidious organisms, we opted to use the gradient-test (GT) in Columbia Blood Agar as an alternative.

In similar fashion to previous studies by Michael O. Alberti et al., where they compared categorical agreement (CA) and essential MIC agreement (EA) between the CLSI BMD reference method and E-tests using a commercially available Chocolate Mueller Hinton Agar (CMHA) plate for vancomycin, PEN, and CRO [32,33], we also observed poor agreement between the two methods. In our case, this was seen to a lesser extent for PEN (BMD MIC 0.125 mg/L vs. GT MIC 0.38–0.5 mg/L) but more significantly for CRO (BMD MIC 0.094 mg/L vs. GT MIC 32 mg/L) and cefotaxime (BMD MIC 0.125 mg/L vs. GT MIC 8–12 mg/L).

In our experience, using the gradient-cross method to test for synergy (through FIC index), we observed a clear synergistic effect with double beta-lactam treatment, specifically with AMP plus BPR and AMP plus CRO at their MICs (despite CRO being non-susceptible by GT). We hypothesize that the basis for this synergistic activity may be due to the differential and complementary saturation of PBPs (similarly to *E. faecalis*), resulting in a necessary bactericidal effect. In order to confirm this topic, we have already started to study the possible *G. adiacens* PBP BPR inhibition, finding promising results concerning interaction with PBP2 and PBP1 family.

Interestingly, our strain showed daptomycin (DAP) nonsusceptibility (DNS) (MIC ≥ 2 mg/L) and internal colonies displayed a high-level DAP resistance (HLDR) profile (MIC ≥ 256 mg/L) based on existing CLSI breakpoints for viridans group streptococci. In a recent Spanish study evaluating DAP susceptibility among nine *G. adiacens* isolates, baseline MICs varied between 1 and 16 mg/L, of which only one (1/9–11.1%) showed a DAP MIC of 1 mg/L. After being incubated with inhibitory concentrations of DAP, all strains rapidly increased the baseline MIC of daptomycin and showed resistance with either a DNS or HLDR profile. Even combination therapy did not prevent the development of DAP resistance with AMP (2/3 strains), CN (2/3 strains), CRO (2/3 strains), or ceftaroline (2/3 strains), thus rising further doubts about the optimal antibiotic treatment for these species [34].

## 5. Conclusions

While *G. adiacens* IE accounts for less than 5% of all streptococci IE cases, it is important to note that, as part of the NVS group, this type of endocarditis has historically been associated with higher rates of morbidity and mortality. This is in part attributed to challenges in culturing and susceptibility testing, which can lead to diagnostic delays and uncertainties in treatment decisions.

Even if, in our case, the surgical intervention was performed very early after the double beta-lactam treatment was started, thus surely leading to better source control and final good clinical outcome, it is equally important to point out that the surgery, as much as it tries to be demolitive, cannot totally eradicate all of the microbiological burden around and into the peri-valvular cardiac tissue. 

Our findings highlight the importance of considering double beta-lactam treatment as a potential option for *G. adiacens* IE, especially in cases where aminoglycosides are contraindicated or not tolerated. It is plausible to consider that the broader saturation of PBPs may contribute to a favorable synergy, enhanced killing, and good clinical outcome, similar to *E. faecalis*, although this theory is still speculative and requires further confirmation.

## Figures and Tables

**Table 1:** *Granulicatella adiacens* MIC Values by E-test (mg/L).

	PEN	AMP	AML	BPR	CPT	CRO	CTX	IMI	LNZ	CN	STR	TEI	VAN	DAP	TGC	DAL
	NS	NS	S	NA	NA	R	R	S	NA	NA	NA	NA	S	NA	NA	NA
*Granulicatella*	0.5	0.5	0.125	0.19	2	32	8	0.25	2	4	1	1	1	4 *	0.094	0.125

S: Susceptible; NS: Non-Susceptible; R: Resistant; NA: Not applicable. PEN: Penicillin; AMP: Ampicillin; AML: Amoxicillin; BPR: Ceftobiprole; CPT: Ceftaroline; CRO: Ceftriaxone: CTX: Cefotaxime: IMI: Imipenem; LNZ: Linezolid; CN: Gentamicin; STR: Streptomycine; TEI: Teicoplanin; VAN: Vancomycin; DAP: Daptomycin; TGC: Tigecycline; DAL: Dalbavancyn. * Internal colonies MIC > 256 mg/L.

**Table 2:** Gradient-Cross Method.

DRUG A	DRUG B	MIC Drug A	MIC Drug B	MIC Drug A in Combination	MIC Drug B in Combination	FIC Drug A	FIC Drug B	FIC Index	INTERPRETATION
AMP 1×	BPR 1×	0.5	0.19	0.125	0.047	0.25	0.247	0.49	SYN
AMP 1×	BPR 2×	0.5	0.38	0.125	0.023	0.25	0.06	0.31	SYN
AMP 1×	CRO 1×	0.5	32	0.094	2	0.188	0.062	0.25	SYN
AMP 1×	CRO 2×	0.5	64	0.047	8	0.094	0.125	0.21	SYN
AMP 1×	CN 1×	0.5	4	0.25	3	0.5	0.75	1.25	IND
AMP 2×	CN 1×	1	4	0.25	0.75	0.25	0.188	0.4	SYN
BPR 1×	CN 1×	0.19	4	0.125	3	0.658	0.75	1.4	IND
BPR 2×	CN 1×	0.38	4	0.094	1	0.247	0.25	0.4	SYN
CRO 1×	CN 1×	32	4	16	2	0.5	0.5	1	ADD
CRO 2×	CN 1×	64	4	4	0.75	0.0625	0.187	0.25	SYN

AMP: Ampicillin; BPR: Ceftobiprole; CRO: Ceftriaxone; CN: Gentamicin. SYN: Synergy; ADD: Additive effect; IND: Indifferent.

## Data Availability

The original contributions presented in the study are included in the article, further inquiries can be directed to the corresponding author.

## List of Abbreviations

NVS	Nutritionally variant streptococci
IE	Infective endocarditis
NVE	Native valve endocarditis
PVE	Prosthetic valve endocarditis
AHA	American Heart Association
ESC	European Society of Cardiology
GL	Guidelines
PEN	Penicillin
AMP	Ampicillin
BRP	Ceftobiprole
CRO	Ceftriaxone
AML	Amoxicillin
CN	Gentamicin
AG	Aminoglycoside
PBPs	Penicillin-binding proteins
PM	Pacemaker
ED	Emergency department
CRP	C-reactive protein
CT	Computed tomography
TTE	Transthoracic echocardiography
MIC	Minimum inhibitory concentration
MBC	Minimum bactericidal concentration
MBIC	Minimum biofilm inhibitory concentration
BMD	Broth micro-dilution
FIC index	Fractional inhibitory concentration index
AST	Antimicrobial susceptibility testing
EFIE	Enterococcus faecalis infective endocarditis
CMHA	Chocolate Mueller Hinton Agar
